# 

**Published:** 1911-11

**Authors:** 


PUBLISHED MONTHLY. ATLANTA SUBSCRIPTION $1.00

### ATLANTA JOURNAL-RECORD OF MEDICINE

LIBRARY
DEC. 6 1911
SURGEON-GENERAL'S OFFICE

Successor to { Atlanta Medical and Surgical Journal, Established 1855. and Southern Medical Record, Established 1870. } 57th Year

Vol. LVII. Atlanta, Ga., November 1911. No. 8

